# The Molecular Mechanism of Multiple Organ Dysfunction and Targeted Intervention of COVID-19 Based on Time-Order Transcriptomic Analysis

**DOI:** 10.3389/fimmu.2021.729776

**Published:** 2021-08-24

**Authors:** Miao Zou, Xiaoyun Su, Luoying Wang, Xingcheng Yi, Yue Qiu, Xirui Yin, Xuan Zhou, Xinhui Niu, Liuli Wang, Manman Su

**Affiliations:** Department of Regenerative Medicine, School of Pharmaceutical Sciences, Jilin University, ChangChun, China

**Keywords:** COVID-19, TO-GCN, cytokine storm, multiple organ dysfunction, targeted intervention, BFS algorithm, multiple organ heterogeneity

## Abstract

Coronavirus disease 2019 (COVID-19) pandemic is caused by the novel coronavirus that has spread rapidly around the world, leading to high mortality because of multiple organ dysfunction; however, its underlying molecular mechanism is unknown. To determine the molecular mechanism of multiple organ dysfunction, a bioinformatics analysis method based on a time-order gene co-expression network (TO-GCN) was performed. First, gene expression profiles were downloaded from the gene expression omnibus database (GSE161200), and a TO-GCN was constructed using the breadth-first search (BFS) algorithm to infer the pattern of changes in the different organs over time. Second, Gene Ontology enrichment analysis was used to analyze the main biological processes related to COVID-19. The initial gene modules for the immune response of different organs were defined as the research object. The STRING database was used to construct a protein–protein interaction network of immune genes in different organs. The PageRank algorithm was used to identify five hub genes in each organ. Finally, the Comparative Toxicogenomics Database played an important role in exploring the potential compounds that target the hub genes. The results showed that there were two types of biological processes: the body’s stress response and cell-mediated immune response involving the lung, trachea, and olfactory bulb (olf) after being infected by COVID-19. However, a unique biological process related to the stress response is the regulation of neuronal signals in the brain. The stress response was heterogeneous among different organs. In the lung, the regulation of DNA morphology, angiogenesis, and mitochondrial-related energy metabolism are specific biological processes related to the stress response. In particular, an effect on tracheal stress response was made by the regulation of protein metabolism and rRNA metabolism-related biological processes, as biological processes. In the olf, the distinctive stress responses consist of neural signal transmission and brain behavior. In addition, myeloid leukocyte activation and myeloid leukocyte-mediated immunity in response to COVID-19 can lead to a cytokine storm. Immune genes such as *SRC*, *RHOA*, *CD40LG*, *CSF1*, *TNFRSF1A*, *FCER1G*, *ICAM1*, *LAT*, *LCN2*, *PLAU*, *CXCL10*, *ICAM1*, *CD40*, *IRF7*, and *B2M* were predicted to be the hub genes in the cytokine storm. Furthermore, we inferred that resveratrol, acetaminophen, dexamethasone, estradiol, statins, curcumin, and other compounds are potential target drugs in the treatment of COVID-19.

## Introduction

Coronavirus disease 2019 (COVID-19) is caused by an infectious, occult, and lethal novel coronavirus, which has led to an ongoing worldwide pandemic (1). It obtains new peculiarities through continuous variation and causes acute respiratory distress syndrome and multiple organ dysfunction that seriously endanger the health of the patient (2). Angiotensin-converting enzyme 2 (ACE2) plays a major role in the renin–angiotensin system (RAS) regulatory pathway and is the main protein that severe acute respiratory syndrome coronavirus 2 (SARS-CoV-2) binds to in the host cells (3, 4). After SARS-CoV-2 combines with ACE2, the protease transmembrane protease serine 2 (TMPRSS2) is activated by the viral spike protein, resulting in various degrees of pulmonary interstitial fibrosis; intestinal, esophageal, and gastric mucosa degeneration; necrosis; abscissions; and other acute injuries (5). This can also cause hepatocyte degeneration, glomerular congestion, segmental hyperplasia or necrosis, cerebral congestion, edema, degeneration, and ischemic changes in neurons (6–8). Briefly, SARS-CoV-2 infection leads to different degrees of injury and diverse clinical symptoms in different organs.

Researchers have found that a cytokine storm is the main cause of severe illness in SARS-CoV-2 (9). Cytokines are the early warning signals when pathogens enter the body, triggering an immune system response to attack them (10). Saha et al. found that after SARS-CoV-2 invades the body, a large amount of self-replication induces the host cells to produce and release interleukin-1 (IL-1), interleukin- 6(IL-6), tumor necrosis factor α(TNF-α), and other interleukin-based cytokines (11). Interleukins dilate the endodermis of adjacent blood vessels, increasing their permeability and triggering a cytokine storm that destroys type I and II alveolar cells, eventually destroying the entire alveoli (12, 13). The loss of type I and type II cells results in dysfunctional gas exchange in the respiratory system. Excess interleukins are transported through blood vessels to the hypothalamus, guiding it to reset the body temperature to a higher level (14). In the case of severe pulmonary inflammation, excessive interleukin can even penetrate the bloodstream and distribute throughout the body, stimulating the body to produce systemic inflammatory response syndrome, which may eventually lead to septic shock (15). Cytokine storm is a clinical feature of systemic inflammatory response failure (16, 17); however, its underlying molecular mechanism is still unclear.

Clinical studies have shown that bronchial transient secretory cells (18), nasal secretory cells, alveolar epithelial cells, brain cells, and intestinal epithelial cells are the main sources of SARS-CoV-2 detection (19), and are also the main target organs of SARS-CoV-2 attack. At the transcriptome level, the present study explored the key mechanisms by which SARS-CoV-2 causes dysfunction in multiple organs, including the brain, lungs, trachea, olfactory bulb (olf), and small intestine (smint). We constructed a time-order gene co-expression network (TO-GCN) to analyze the dynamic changes in gene expression and different biological processes in various organs after being infected by SARS-CoV-2. The hub genes of each organ were identified, and compounds targeting the hub genes were identified as potential targeted drugs for COVID-19.

## Materials and Methods

**Figure 1:**
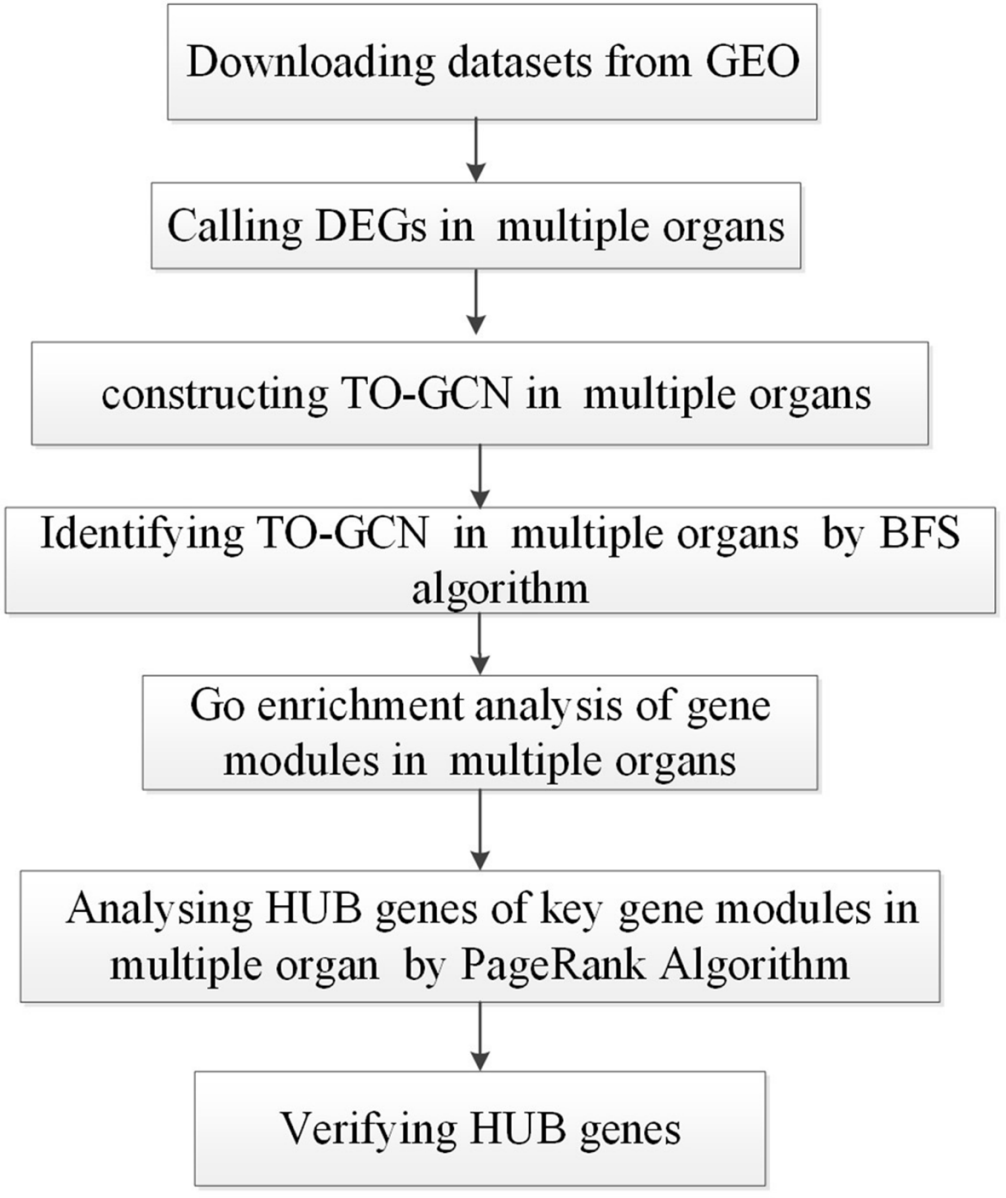
The overall flow chart of the data analysis conducted in this study.

The TO-GCN analysis method was applied to detect the changes in the gene expression profiles of five organs, namely the brain, lung, trachea, olf, and smint, on days 0, 1, 2, 4, 6, 8, and 14 days after SARS-CoV-2 infection in Syrian golden hamsters (*Mesocricetus auratus*). **Figure 1** shows the flow chart of the study design.

### Data Preprocessing

The gene expression profiles of the five organs of Syrian golden hamsters infected with a low dose of SARS-CoV-2 on days 0, 1, 2, 4, 6, 8, and 14 were obtained from the GEO database (https://www.ncbi.nlm.nih.gov/geo/, GSE161200). Five samples of normal control groups and three samples of experimental groups were tested in each subgroup. A total of 22,283 genes were sequenced in this study.

At first, we converted the read counts to counts per million (CPM) values to standardize the data sets. Then, zero-expressed genes were removed to complete the data preprocessing (**Supplementary Table 1**). The CPM calculation was implemented using the edgeR package in R version 3.28.1 (20).

### Identification of Differentially Expressed Genes (DEGs)

In our study, the DEseq2 algorithm was selected to identify significant DEGs (21, 22). The fold change threshold was set at log2 (fold change) > log2(1.5) or log2 (fold change) < -log2(1.5), and p-adj was set as p-adj < 0.05. The gene expression profiles of five organs on days 1, 2, 4, 6, 8, and 14 after SARS-CoV-2 infection were compared with the normal control group (day 0) to identify the differences in gene expression in each organ, and all the differences in gene expression were combined to obtain the DEGs in each organ for further analysis. DEGs were screened using the DEseq2 function in R (version 3.6.3) (23, 24).

### Construction of TO-GCN

The Pearson’s correlation coefficient (PCC) is an important index for determining the strength of synergistic or antagonistic action between genes (25). Considering the transcriptome data of DEGs at six time points as the background data, the Pearson’s correlation algorithm was selected to calculate the correlation coefficients between two genes in five organs, and the PCC and *p* value were obtained. The cut-off value was set as PCC > 0.75 and *p* < 0.05 to screen the interaction relationship between genes. TO-GCN was established by co-expressing genes that met the threshold requirements.

### Time-Order Gene Modules Identification Using the Breadth-First Search (BFS) Algorithm

In TO-GCN, the two interlinked genes showed similar changes over time (26). The BFS algorithm was applied to infer the temporal expression order of all genes in the TO-GCN (27). First, the genes with peak gene expression at the first time point and decreasing trend over time were defined as the initial node of TO-GCN. Next, the BFS algorithm was selected to traverse the TO-GCN to infer the gene rule of dynamic changes and obtain time-order gene modules. Finally, we used Gene Ontology (GO) enrichment analysis to biologically annotate the time-order gene modules.

### Construction of Key Gene Modules

The initial gene modules involved in the immune response of different organs were selected as the research objects to elucidate the key mechanism of the immune response of the body (28). The NCBI database (https://ftp.ncbi.nlm.nih.gov/gene/DATA/) was used to map the human genes in these modules that are homologous to the Syrian golden hamster. Furthermore, the ImmPort database (https://www.immport.org) was used to identify immune-related genes in the modules (29, 30). Finally, the genes were imported into the STRING database (https://string-db.org/) to construct immune protein–protein interaction (PPI) networks for different organs (31). Multiple proteins were selected in the “names/identifiers” option in the query mode, and the minimum required interaction score was set to “medium confidence” (0.400).

### Identification of Hub Genes

The PageRank algorithm was proposed by Larry Page and Sergey Brin, the founders of Google, to calculate the importance of nodes in a complex network (32). It was used to score the importance of genes in the PPI network (based on topological principles) (33). We defined five genes with the highest scores in each organ as hub genes.

### Verification of Hub Genes

First, GSE166253 and GSE162615 were downloaded from the GEO database to identify the mRNA expression levels of hub genes. There were 10 normal samples (GSM5066812-GSM5066821), six COVID-19 recovery samples (GSM5066822-GSM5066827), and 10 COVID-19 retesting-positive samples (GSM5066828-GSM5066837) in GSE166253. GSE162615 consisted of 18 normal and COVID-19 samples (GSM4955401-GSM4955418). Furthermore, t-tests were used to verify the differences in hub genes.

### Screening the Potential Targeted Drugs

Using the Comparative Toxicogenomics Database (CTD) (https://ctdbase.org/), which contains chemical and gene interactions, potential drugs that target the hub genes in the five organs were investigated.

The selected hub genes were imported into the CTD database and classified according to the interaction relationship between genes and compounds. The 10 compounds with the highest scores were identified as potential target compounds of hub genes.

## Results

### COVID-19 Caused Changes in Multiple Organs at Transcriptome Levels

The data on day 0 of SARS-CoV-2 infection were used as the normal control group, and the data of days 1, 2, 4, 6, 8, and 14 were used as the experimental group. To understand the changes in gene expression related to multiple organ dysfunction after being infected by SARS-CoV-2, we used the DESeq2 algorithm to screen DEGs of the five organs. DEG distribution results of different organs are shown in **Supplementary Figure 1** and **Supplementary Table 2**. The results showed that the expression of different genes in multiple organs had an obvious time phase.

**Figure 2:**
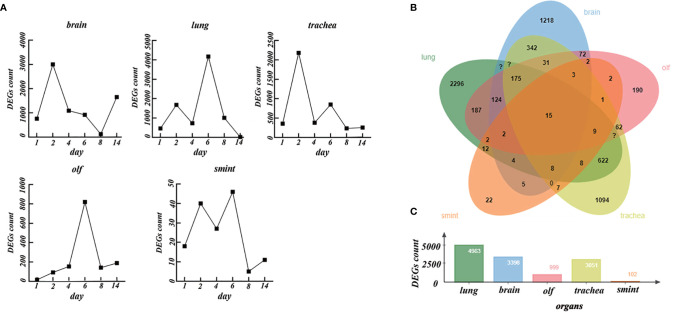
The differentially expressed genes (DEGs) in different organs over time. **(A)** The number of DEGs in the brain, lung, trachea, olf, and smint over time. **(B)** Venn diagrams of DEGs in the brain, lung, trachea, olf, and smint. **(C)** The value of total DEGs in each organ.

As shown in **Figure 2A**, on day 1 after the brain was infected by SARS-CoV-2, 507 DEGs (*NDRG2*, *RPSA*, *TRF*, etc.) were upregulated, and 254 DEGs (*COL4A2*, *RGS5*, *ATP13A5*, etc.) were downregulated. On day 2, 1,562 DEGs (*MX2*, *IRF9*, *TRF*, etc.) were upregulated, and 1,438 DEGs (ENSMAUG00000008753, ENSMAUG00000014490, etc.) were downregulated. Subsequently, the number of DEGs decreased gradually. On days 4, 6, and 8, the number of upregulated DEGs was 660 (*TRF*, *AGT*, etc.), 439 (*TRF*, *AGT*, etc.), and 88 (*PYGB*, *OGDHAND*, etc.), whereas the number of downregulated DEGs was 430 (*COL4A2*, etc.), 478 (ENSMAUG00000019419, etc.), and 37 (*SRSF5*, etc.), respectively. Notably, on day 14, the differential expression levels rebounded (910 upregulated and 745 downregulated).

In the lung, the number of DEGs increased rapidly and lasted for a relatively long time. On days 1, 2, 4, and 6, the number of upregulated DEGs was 90 (RNF5, etc.), 1,000 (*IRF7*, *MX2*, etc.), 329 (*IRF7*, *MX2*, etc.), and 2,154 (*IRF7*, *MX2*, etc.), whereas the number of downregulated DEGs was 369 (*Pcnp*, *CACRL*, *Serp1*, etc.), 675 (ENSMAUG00000011900, ENSMAUG00000017566, etc.), 398 (*DBP*, etc.), and 2,020 (*CALCRL*, etc.), respectively. The number of DEGs decreased rapidly on day 8. There were 477 upregulated DEGs (*IRF7*, *MX2*, etc.) and 599 downregulated DEGs (ENSMAUG00000001317, ENSMAUG00000011754, etc.) on day 8. On day 14, the DEGs almost disappeared.

In the trachea, there were 158 upregulated DEGs (*SLC16A11*, *UCP2*, etc.) and 198 downregulated DEGs (*RNF112*, *RHCG*, etc.) on day 1. However, the number of DEGs increased significantly on day 2 compared to that on day 1. There were 1,315 upregulated DEGs (IRF7, etc.) and 867 downregulated DEGs (ENSMAUG00000000032, ENSMAUG00000001101, etc.) on day 2, and then, the number of DEGs decreased gradually. On days 4 and 6, the number of upregulated DEGs was 290 (*IRF7*, etc.) and 653 (*IRF7*, etc.), whereas the number of downregulated DEGs was 653 (*RNF112*, etc.) and 195 (ENSMAUG00000011132, etc.). The DEGs decreased persistently on days 8 and 14.

In the olf, the number of DEGs increased rapidly and lasted for a relatively long time. On days 1, 2, 4, and 6, the number of upregulated DEGs was 13 (*SLC3A1*, *MX2*, etc.), 71 (*MX2* and *MX1*), 128 (*MX2*, *B2M*, etc.), and 548 (*SERPING*, *MX2*, etc.), whereas the number of downregulated DEGs was 3 (*FOSL2*, *NR4A2*, etc.), 21 (*FOSL2*, *NR4A2*, etc.), 25 (ENSMAUG00000013627, ENSMAUG00000007358, etc.), and 272 (ENSMAUG00000019419, ENSMAUG00000021143, etc.), respectively. However, it rapidly decreased on day 8 to 62 (*C3*, *B2M*, etc.) upregulated DEGs and 79 (*FOSL2*, etc.) downregulated DEGs. On day 14, there were 162 upregulated DEGs (*ND6*, *SLC3A1*, etc.) and 26 downregulated DEGs (*GNAS*, etc.). There were few DEGs in the smint. The number of upregulated genes was 2 (ENSMAUG00000017595 and ENSMAUG00000012989), 31(*UBA7*, *MX2*, etc.), 11(*UBA7*, etc.), and 10 (ENSMAUG00000021358, ENSMAUG00000017595, etc.), and the number of downregulated DEGs was 16 (*COL18A1*, *COX1*, etc.), 9 (ENSMAUG00000003218, ENSMAUG00000000032, etc.), 16 (*NR1D1*, etc.), and 36 (*JCHAIN*, etc.) on day 1, 2, 4, and 6, respectively. The DEGs almost disappeared on day 8. Except for the brain and trachea (on day 2), peak values of DEGs were observed on day 6 in the smint, lung, and olf.

Next, we considered a union of the DEGs of each organ at different time points. There were total of 3,398, 4,983, 3,051, 999, and 102 DEGs in the brain, lung, trachea, olf, and smint, respectively (**Figures 2B, C**). Despite the significant differences in DEGs in the five organs, 15 genes that were primarily involved in the immune system response (interferon α/β signaling) and immune system cytokine signaling were significantly differentially expressed in all the five organs after SARS-CoV-2 infection compared with the normal control samples (**Figure 2B**). Based on the abovementioned results, we inferred that the dynamic changes in gene expression in the lungs, trachea, olf, and smint after being infected by COVID-19 were similar, but the dynamic changes in the brain were quite different from those in the other organs. The regulation of immune system reactions may be an important mechanism for multiple organ dysfunction.

### Construction of Organ-Specific TO-GCN After SARS-CoV-2 Infection

The number of DEGs in smint (102) was too small to be analyzed from the perspective of TO-GCN. The DEGs transcriptome data of the brain, lung, trachea, and olf were used as background data to investigate the genetic mechanisms of the dynamic changes in multiple organs after SARS-CoV-2 infection.

**Figure 3:**
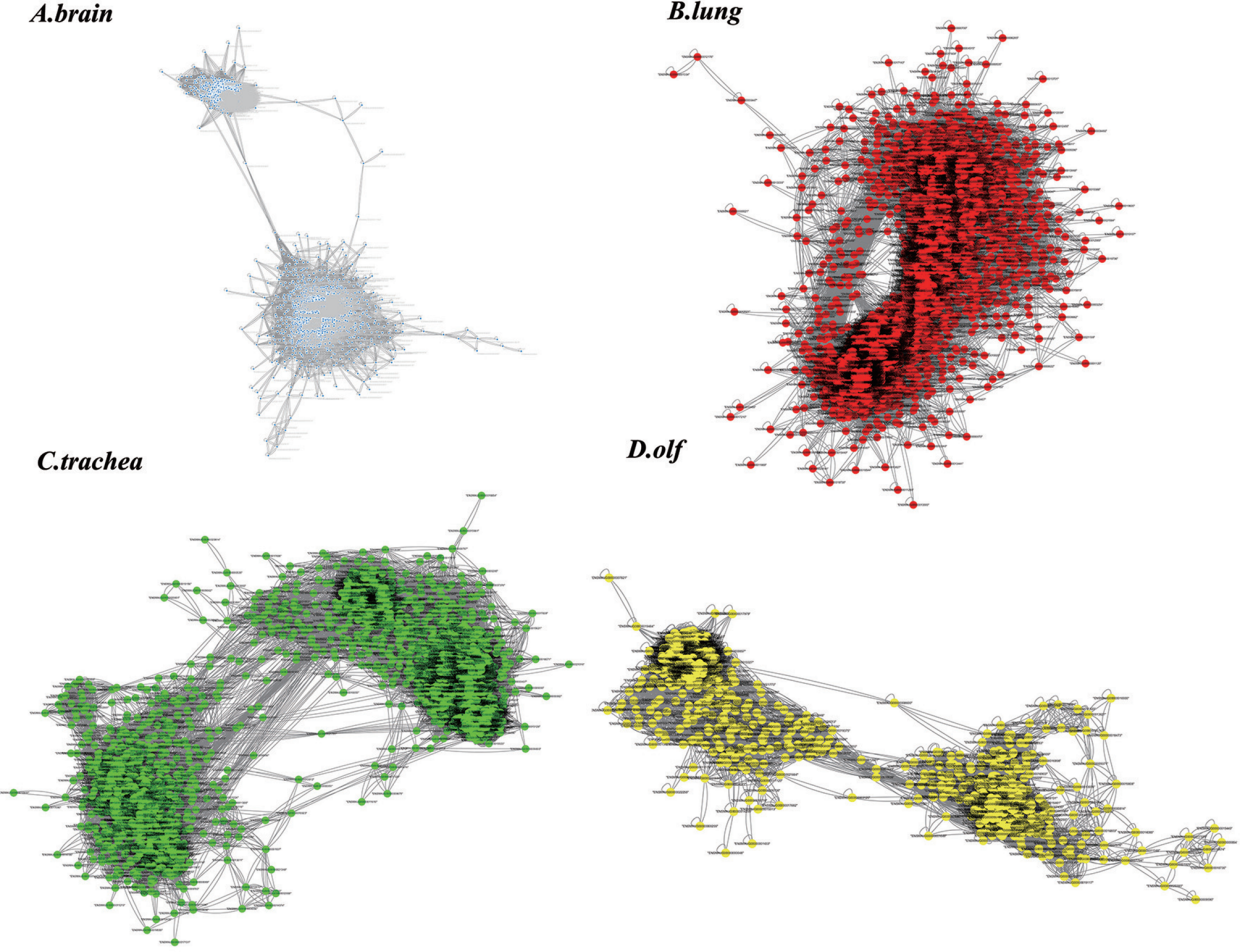
The results of the time-order gene co-expression network (TO-GCN) analysis in the **(A)** brain, **(B)** lung, **(C)** trachea, and **(D)** olf. The colored dots represent the screened genes.

The Pearson’s correlation analysis was used to construct a TO-GCN for each organ, including the brain, lung, trachea, and olf, and genes that met the threshold criteria (PCC > 0.75 and p-adj < 0.05) were screened. Next, the sub-networks with fewer than 20 genes were removed, and the network with the largest number of genes was defined as a TO-GCN (**Figure 3**).

### GO Analysis of TO-GCN After SARS-CoV-2 Infection

**Figure 4:**
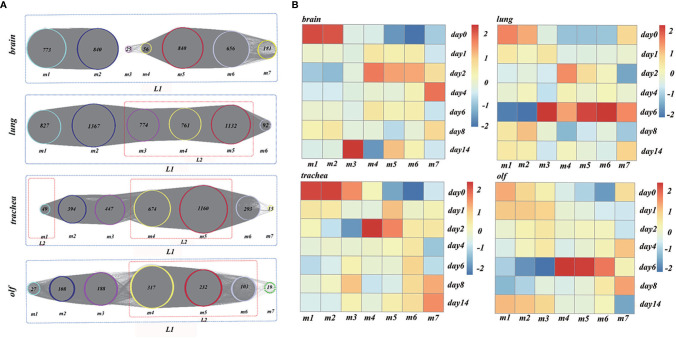
**(A)** The analysis results of time-order gene modules in different organs. L1 represents the body’s stress response, whereas L2 represents the immune response mediated by the immune cells. **(B)** Heatmaps of the average normalized counts per million (CPMs) in different organs.

The genes with peak gene expression at the first time point and a decreasing trend over time were defined as the initial node of TO-GCN. ENSMAUG00000021998, ENSMAUG00000022214, ENSMAUG00000010844, and ENSMAUG00000012900 were selected as the initial nodes of the brain, lung, trachea, and olf, respectively. Then, we used the BFS algorithm to traverse the TO-GCNs to infer the time-order gene modules of different organs. As shown in **Figure 4A**, there were six to seven time-order gene modules that were all involved in the stress response in each organ. Except for the brain, there were time-order gene modules related to immune responses mediated by immune cells in the lung, trachea, and olf.

To establish the reliability of the time-order analysis results, we constructed a heatmap for each organ based on the gene expression data of the time-order gene modules over time (**Figure 4B**). It was found that the gene expression of time-order gene modules roughly presented a main diagonal distribution in different organs.

### Multiple Organ Heterogeneity Based on the TO-GCN After SARS-CoV-2 Infection

**Figure 5:**
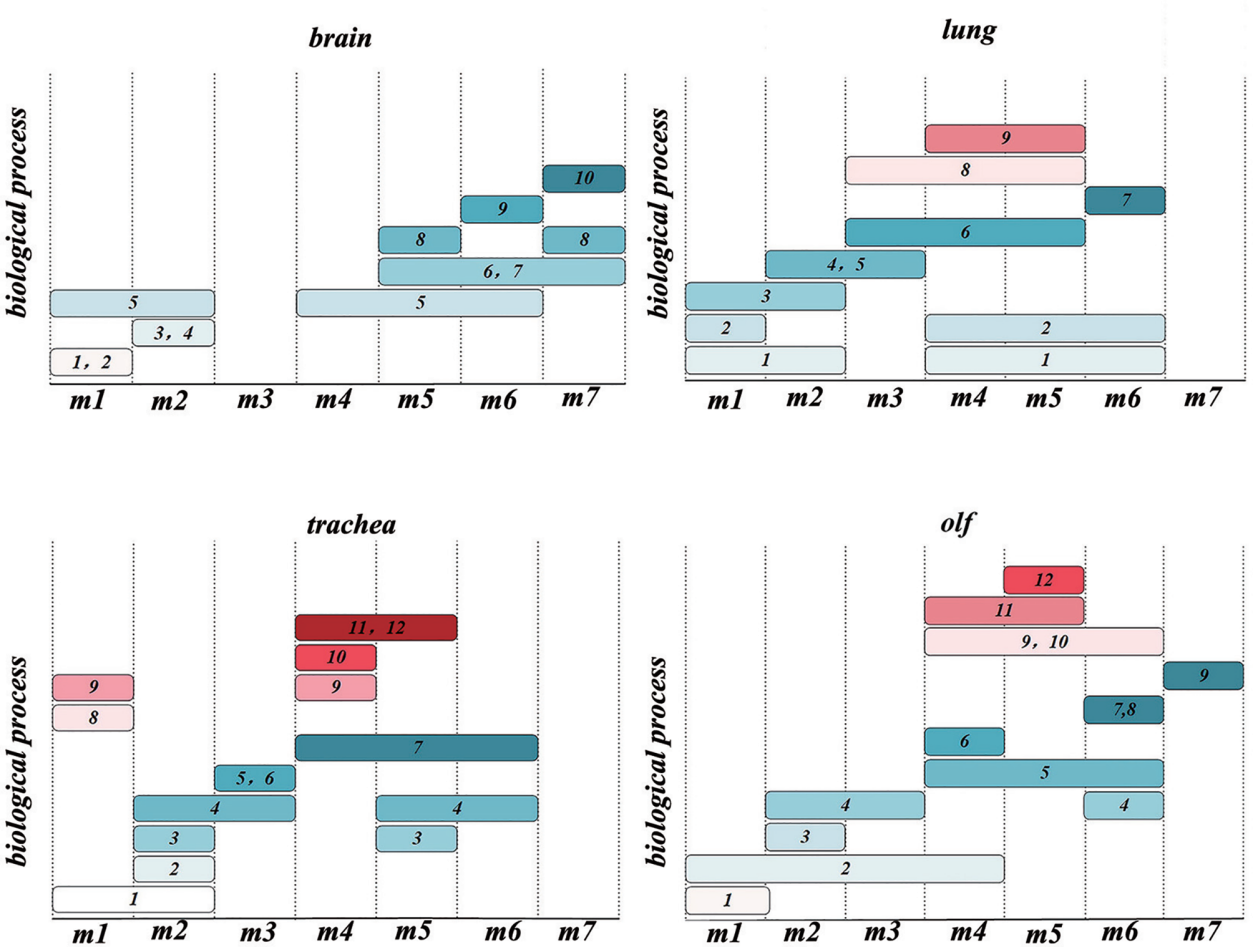
Time-order Gene Ontology (GO) enrichment analysis results of different organs 1. nucleic acid metabolic process; 2. cell cycle; 3. nervous system process; 4. signal transduction; 5. cellular component organization; 6. energy derivation by oxidation of organic compounds; 7. ATP metabolic process; 8. nucleoside phosphate metabolic process; 9. protein metabolic process; 10. ion transmembrane transport; 11. regulation of locomotion; 12. vasculature development; 13. secretion by cell; 14. lipid metabolic process; 15. myeloid leukocyte activation; 16. myeloid leukocyte-mediated immunity; 17. macromolecule localization; 18. protein catabolic process; 19. cellular metabolic process; 20. nucleotide metabolic process; 21. muscle system process; 22. response to stimulus; 23. immune response-regulating signaling pathway; 24. regulation of innate immune response; 25. antigen processing and presentation; 26. head development; 27. small molecule metabolic process; 28. vesicle-mediated transport; 29. regulation of body fluid levels.

To explore the multiple organ heterogeneity of biological processes after the organs were infected by SARS-CoV-2, the biological significance of each organ in time-order gene modules was explored using the GO enrichment analysis (**Supplementary Table 3**). As shown in **Figure 5**, there are three stages of biological processes in the brain: the first stage (m1) mainly affected the regulation of the cell cycle, mitosis (mitotic cell cycle phase transition, etc.), and chromosome composition (cilium organization, regulation of chromosome organization, etc.). The second stage (m2) mainly affected the neuron development (positive regulation of neuron projection development and cell morphogenesis involved in neuron differentiation) and synaptic signaling (anterograde trans-synaptic signaling, chemical synaptic transmission, synaptic signaling, etc.). The third stage (m4–m7) mainly affected the mitochondrial translational elongation (ATP metabolic process, etc.). There were four stages in the lung: the first stage (m1) mainly affected the cellular tissue components (extracellular matrix organization, proteolysis involved in cellular protein catabolic process, etc.). The second stage (m2) mainly affected cell migration (positive regulation of locomotion, positive regulation of cellular component movement, etc.) and the regulation of angiogenesis (blood vessel morphogenesis, angiogenesis, etc.). The third stage (m3–m5) mainly affected the myeloid leukocyte-mediated immunity (myeloid leukocyte activation). The fourth stage (m6) mainly affected the regulation of mitochondrial-related energy metabolism (mitochondrial translational elongation, mitochondrial translational termination, etc.). There were four stages in the trachea: the first stage (m1) mainly affected the regulation of innate immune response (innate immune response-activating signal transduction, activation of innate immune response, etc.). The second stage (m2, m3) mainly affected the regulation of biological processes related to protein metabolism (ubiquitin-dependent protein catabolic process, proteolysis involved in cellular protein catabolic process, etc.) and rRNA metabolism (ribonucleotide metabolic process, purine ribonucleotide metabolic process, etc.). The third stage (m4, m5) mainly affected the myeloid leukocyte-mediated immunity (myeloid leukocyte activation) and antigen processing and presentation. The fourth stage (m6) was the oxidative stress response. There were three stages in the olf. The first stage (m1) mainly affected the regulation of brain behavior-related biological processes (learning or memory, behavior, brain development, etc.). The second stage (m2, m3) mainly affected the regulation of synaptic plasticity (cell morphogenesis involved in neuron differentiation, etc.). The third stage (m4–m6) mainly affected the same biological processes as the third stage in the trachea and lung.

Therefore, there are two types of biological processes: the stress response and immune cell-mediated immune response involving the lungs, trachea, and olf after SARS-CoV-2 infection. However, a single biological process of stress response occurs in the brain, and the stress response is heterogeneous in different organs. In the lung, the regulation of DNA morphology, angiogenesis, and mitochondrial-related energy metabolism are specific biological processes related to the stress response. Biological processes related to the regulation of protein and rRNA metabolism-related biological processes are the main stress responses of the trachea. In olf, the distinctive stress response is neural signal transmission and brain behavior. It is worth mentioning that the myeloid cell-mediated immune response-related biological processes caused by SARS-CoV-2 infection occurred in the lungs, olf, and trachea. Neuron morphogenesis caused by SARS-CoV-2 infection mainly occurs in the nervous system, such as in the brain and olf.

These results demonstrated that the immune responses in the trachea, lung, and olf were in different modules; however, the time of immune response occurrence was consistent with the time when the number of DEGs peaked in different organs.

### Hub Genes of the Key Gene Modules in Multiple Organs After Being Infected by SARS-CoV-2

Zhou et al. found that there were two clinical stages of SARS-CoV-2 infection in the human body. The first stage is the initial incubation stage, which is highly infectious, but with less obvious clinical symptoms. The second stage is the later clinical symptoms stage, when the immune response is excessively activated and the inflammatory reaction causes multiple organ injuries (34). In our study, 15 genes that were significantly differentially expressed in the lungs, olf, and trachea were found to be involved in the immune response. GO enrichment analysis of the time-order gene modules showed that the immune response mediated by immune cells occurred in the lungs, trachea, and olf. Based on the abovementioned results, we speculated that over-activation of the immune response was not only an important marker of clinical symptoms but also a key mechanism of SARS-CoV-2-induced multiple organ dysfunction (34). Therefore, the gene modules of the immune response were used to explore the hub genes that mediate the immune response in different organs after SARS-CoV-2 infection.

**Figure 6:**
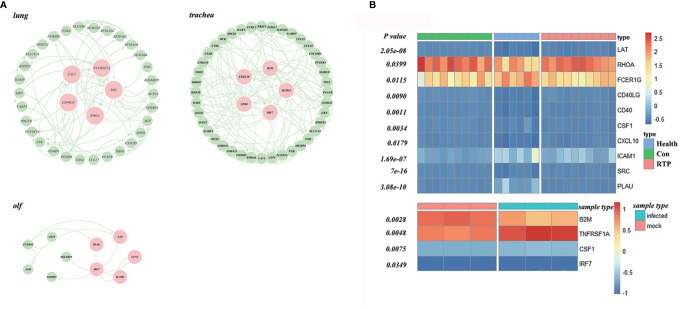
Screening and validation of hub genes in multiple organs after SARS-CoV-2 infection. **(A)** Hub genes in the protein–protein interaction (PPI) network screened using the PageRank algorithm (Red ones indicate the hub genes). **(B)** Validation of hub genes based on datasets GSE166253 and GSE162615, GSE166253 and GSE162615.

First, the ImmPort database was used to identify the genes related to immune response, and the STRING database was used to construct a PPI network. Finally, the PageRank algorithm was used to identify hub genes in the lungs, trachea, and olf (**Supplementary Table 4**). Among them, SRC, RHOA, CD40LG, CSF1, and TNFRSF1A were the hub genes in the lung; hub genes in the trachea were CXCL10, ICAM1, CD40, IRF7, B2M, whereas FCER1G, ICAM1, LAT, LCN2, and PLAU were the hub genes of olf (**Figure 6A**).

In this study, GSE166253 and GSE162615 datasets were used as background data, and the t-test was used to verify the hub genes. (**Figure 6B**). The expression of all the hub genes mentioned above was significantly different between the COVID-19 and normal control groups.

### Screening Targeted Drugs Based on the Hub Genes

**Figure 7:**
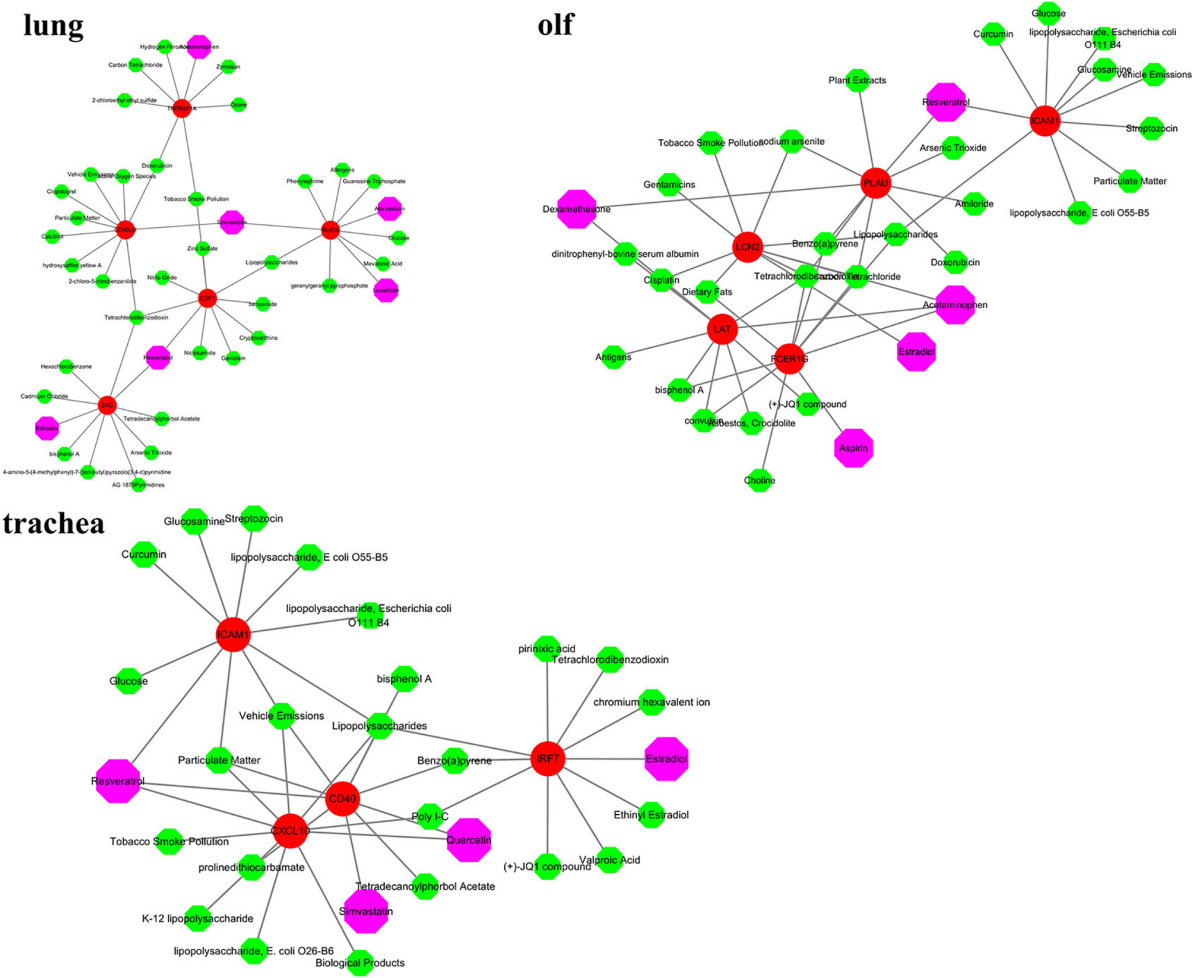
Screening of potential therapeutic drugs for different organ dysfunctions. Red indicates hub genes of organs, whereas purple indicates potential therapeutic drugs that target hub genes.

The hub genes were used as targets to explore potential drugs for COVID-19 treatment using the CTD database (**Supplementary Table 5**). As shown in **Figure 7**, compounds such as resveratrol, acetaminophen, estradiol, statins, dexamethasone, and quercetin are potential targeted drugs for managing COVID-19. Resveratrol and estradiol were screened as they could regulate the expression of multiple genes such as *SRC*, *CSF1*, and *ICAM10* in the lung, trachea, and olf after SARS-CoV-2 infection. Acetaminophen might have a therapeutic effect on lung and olfactory dysfunction by regulating the expression of *TNFRSF1A*, *FCER1G*, and *LAT*. Statins had therapeutic effects on lung and tracheal dysfunction by regulating the expression of *RHOA*, *CD40LG*, and *CD40*. Dexamethasone and quercetin had therapeutic effects on olf and tracheal dysfunction by regulating the expression of *LAT*, *PLAU*, *CXCL10*, and *CD40*.

## Discussion

COVID-19 is caused by SARS-CoV-2, which primarily affects the lungs and gradually spreads to multiple organs, leading to multiple organ dysfunction (35). However, the mechanisms underlying multiple organ dysfunction remain unclear. In our study, it was found that the lung, trachea, and olf after SARS-CoV-2 infection mainly demonstrated two biological processes: stress response and immune cell-mediated immune response. However, the stress response is the only biological process in the brain that regulates neuronal signals after SARS-CoV-2 infection. Virhammar et al. first reported a patient with acute necrotizing encephalopathy who was negative for COVID-19. Despite a coma due to neurological deterioration, the patient never experienced a period of severe inflammatory response. Moreover, SARS-CoV-2 RNA, high concentrations of the neuronal injury markers neurofilament light, tau, and astrocyte activation marker glial fibrillary acidic protein were detected in the patient’s cerebrospinal fluid (36). The results of the abovementioned study are consistent with biological processes identified in the brain in our analysis.

In the lung, the regulation of DNA morphology, angiogenesis, and mitochondrial-related energy metabolism are specific biological processes related to the stress response. Belizário et al. found that coronaviruses co-evolved with host cells after pulmonary invasion. The host coordinated the process of recombination, mutation, and repair of coronavirus RNA intermediates while performing RNA editing and DNA repair, which drives the pathological process of COVID-19 (37). Ackermann et al. found that the peripheral pulmonary pathology of COVID-19 patients who died of respiratory failure showed diffuse alveolar injury with perivascular T cell infiltration. Unique vascular features, such as severe endothelial damage and cell membrane destruction associated with the presence of intracellular viruses were observed in the lungs of COVID-19 patients. In addition, extensive thrombosis with microvascular disease has been observed in COVID-19 patients (38). Codo et al. found that the replication of coronavirus in the lungs triggers the production of mitochondrial reactive oxygen species, which stabilizes hypoxia-inducible factor-1α and improves the efficiency of coronavirus replication and glycolysis (36). These studies corroborate our findings regarding the mechanism of pulmonary dysfunction.

In the trachea, biological processes such as the regulation of protein metabolism and rRNA metabolism-related biological processes modulate the stress response. These two biological processes regulate various factors inside and outside the airway smooth muscle to maintain the balance of oxygen exchange in the lung. Airway smooth muscle, a unique type of smooth muscle, forms an effective, adjustable, and reactive wall that covers most of the airway from the trachea to the alveolus. Airway smooth muscle and the surrounding inflammatory environment play important roles in COVID-19 (39). The results showed that after SARS-CoV-2 infection, the trachea responded rapidly to the invading virus through its inherent normal physiological defenses. Eventually, it mediated the initiation and response process of specific immunity.

In the olf, the distinctive stress response was the regulation of biological processes related to neural signal transmission and brain behavior. It has been reported that coronavirus may enter the central nervous system through the olf, triggering cytokine storms and brainstem dysfunction and causing neuronal death (40, 41).

In addition, it was found that the biological processes related to immune response (myeloid leukocyte activation and myeloid leukocyte-mediated immunity) occurred in the lung, olf, and trachea. Immune cells such as neutrophils and mononuclear macrophages can coordinate protective immunity against SARS-CoV-2 infection. However, cytokine storms promote excessive infiltration of immune cells, which cause immune cells to have strong pro-inflammatory activity and cause multiple organ dysfunction (42). Therefore, we conducted a deeper investigation on the effects of the immunity-related genes in various organs after SARS-CoV-2 infection.

In our study, 15 genes were screened as hub genes, which played an important role in the pathological process of COVID-19. In the lungs, COVID-19 causes lung dysfunction by regulating the expression of SRC, RHOA, CD40LG, CSF1, and TNFRSF1A. SRC is a typical member of the non-receptor protein tyrosine kinase family that regulates the activation of T cells (43). Li et al. found that SRC protein tyrosine kinase was expressed by leukocytes, alveolar epithelial cells, endothelial cells, and fibroblasts in the lung. Pulmonary fibrosis is a potentially fatal disease caused by persistent damage to alveolar epithelial cells, resulting in the accumulation of myofibroblasts and excessive deposition of extracellular matrix components and connective tissue. The pro-fibrotic effect of SRC kinase plays an important role in pulmonary fibrosis (44). RHOA, a member of the RHO family of small GTPases that circulates between the inactive GDP-bound state and the active GTP-bound state, acts as a molecular switch in the signal transduction cascade. It is activated during the binding reaction of chemokines, cytokines, and growth factors (45, 46). In addition, Liu et al. found that *RHOA* is the main immune gene that induces lung inflammation and acute lung injury by inhibiting lung cancer cell apoptosis and promoting its proliferation (47, 48). The protein encoded by *CD40LG* is expressed on the surface of T cells. It can effectively regulate the function of B cells. Therefore, CD40LG plays an important role in the conversion of immunoglobulins and the treatment of high IgM syndrome (49, 50). Li et al. found that CD40LG and chemokines are jointly involved in the synthesis of pro-inflammatory mediators, which leads to the occurrence of transfusion-related acute lung injury and autoimmune diseases by attracting leukocytes to the site of inflammation (51). *CSF1* is an immune gene that controls the production, differentiation, and function of macrophages. Bertolazzi et al. found that *CSF1* overexpression after SARS-CoV-2 infection led to pulmonary fibrosis by promoting the recruitment and activation of macrophages (50). TNFRSF1A is a tumor necrosis factor receptor, and the interaction between tumor necrosis factor and TNFRSF1A plays an important role in inhibiting the occurrence of inflammation, tumor proliferation, migration, and invasion (52). Wilson et al. found that TNFRSF1A induced alveolar epithelial cell dysfunction in the early stages of acute respiratory distress syndrome, which promotes lung permeability and inhibits the reabsorption of alveolar fluid (53). In summary, *SRC*, *RHOA*, *CD40LG*, *CSF1*, and *TNFRSF1A* may be the key genes that cause lung dysfunction after the body is infected with coronavirus.

SARS-CoV-2 can damage the trachea by regulating the expression of CXCL10, ICAM1, CD40, IRF7, and B2M. *CXCL10* is an immune gene induced by interferon, which regulates T cell migration by binding to CXCR3. CXCL10 plays an important role in the “cytokine storm” induced by SARS-CoV-2 (54). Belperio et al. found that persistent expression of CXCL10 and CXCR3 led to chronic peribronchiolar leukocyte infiltration, which induced airway fibrous occlusion (55). ICAM1, a cell surface adhesion receptor that regulates the accumulation of white blood cells to inflammation sites, plays an important role in immune cell response in inflammation and tumorigenesis (56). Yamaya et al. found that increased ICAM1 expression levels may lead to increased production of cytokines that induce rhinovirus replication and infection. This biological process induces airway inflammation and aggravates asthma (57). *CD40* encodes a receptor on antigen-presenting cells, which can mediate a variety of immune responses and inflammatory responses (58). Lazaar et al. found that CD40 may mediate an important signal transduction pathway involving protein tyrosine kinase-dependent calcium mobilization, NF-κB activation, and IL-6 production in airway smooth muscle, which interacts with smooth muscle cells to enhance airway inflammation (57, 59, 60). IRF7 can induce the production of interferons (61). Barjesteh et al. found that after chicken tracheal epithelial cells were stimulated by TLR ligands, the IRF7 and NF-κB signaling pathways initiate antiviral responses, which led to the activation of macrophages (62). The mechanism by which B2M deficiency causes primary or acquired drug resistance in immunotherapy affects the normal folding or transport of MHC I to the cell surface and antigen presentation. Detection of immunotreatment-sensitive markers concurrently with possible drug-resistant mutations allows for more accurate screening of immunotherapy methods (63). Cook et al. found that the loss of B2M expression may increase susceptibility to respiratory mycoplasma infection (64). To summarize, the abnormal expression of CXCL10, ICAM1, CD40, IRF7, and B2M may be the main cause of tracheal dysfunction after infection with coronavirus.

SARS-CoV-2 causes damage to the olf and regulates nervous system function by regulating the expression of FCER1G, ICAM1, LAT, LCN2, and PLAU. FCER1G is at the core of allergic reactions. The specific binding of lgE and FCER1G leads to the release of many mediators that can induce allergic reactions (65). Zhang et al. found that the elimination of activated FcγR can regulate nerve damage by changing the endoneurium and systemic inflammation (66). Jahromi et al. found that endothelial cell ICAM-1 and ICAM-2 mediate the migration of Th1 and Th17 cells across the blood–brain barrier in neuroinflammation (67). The synergistic signal transduction system of LAT and SH2 plays an important role in the signal transduction of mast cells and T cell antigen receptors (68). LCN2 plays an important antibacterial function in innate immunity by resisting external stimuli and protecting cells from apoptosis (69). *PLAU* encodes a secreted serine protease. Li et al. found that PLAU exerted a carcinogenic effect in head and neck squamous cell carcinoma by affecting the tumor immune microenvironment (70). In general, *FCER1g*, *ICAM1*, *LAT*, *LCN2*, and *PLAU* may be the main immune genes that are regulated by COVID-19 to induce olf and neurological dysfunction.

The prognosis of COVID-19 is largely influenced by multi-organ response to the novel coronavirus. Cardiovascular disease and multiple organ failure have become the most common risk factors for severe illness and death (71). The CTD database was used to explore potential drugs for hub genes in the lungs, trachea, and olf in our study. Resveratrol and estradiol were screened for their ability to regulate the expression of multiple genes such as *SRC*, *CSF1*, and *ICAM10* in the lung, trachea, and olf after SARS-CoV-2 infection. Resveratrol is a polyphenol compound that has a good preventive effect against many diseases, such as respiratory system disease, cancer, and cardiovascular disease. Pasquereau et al. found that resveratrol has antiviral effects, including on the coronavirus. It stimulates the immune system and inhibits the release of inflammatory cytokines by regulating RAS and upregulating the expression of ACE2 (72–74). Estrogen can effectively regulate the number of immune cells produced by the immune system, which makes women more resistant than men to the cytokine storm induced by SARS-CoV-2 infection and immune system disorders (75). Acetaminophen may have a therapeutic effect on lung and olfactory dysfunction by regulating the expression of TNFRSF1A, FCER1G, and LAT. Acetaminophen, the most classic antipyretic, analgesic, and anti-inflammatory drug, can relieve fever and other symptoms in COVID-19 patients (76). Statins have therapeutic effects on lung and tracheal dysfunction by regulating the expression of RHOA, CD40LG, and CD40. Statins have anti-inflammatory, immunomodulatory, and antithrombotic effects. By upregulating the expression of ACE2, statins minimize lung dysfunction caused by excess angiotensin II (77). Statins restrict the “cytokine storm” in patients with severe COVID-19 by blocking the NF-κB pathway and NLRP3 inflammasomes to exert their anti-inflammatory properties and by reducing the invasion of viruses to destroy lipid rafts (78, 79). In addition, our study found that dexamethasone and quercetin had specific therapeutic effects on olf and tracheal dysfunction by regulating the expression of LAT, PLAU, CXCL10, and CD40. Quercetin exerted a good antiviral effect by inhibiting the expression of DNA gyrase, protease, polymerase reverse transcriptase, and binding to viral capsid proteins. The combination of quercetin and vitamin C has a positive effect on COVID-19 patients (80). Dexamethasone is a broad-spectrum immunosuppressant. Molecular docking studies have shown that dexamethasone inhibits the entry of coronavirus into cells by binding to ACE2 (81). Dexamethasone restricts the production and destruction of inflammatory cytokines, but it also inhibits the protective effects of T cells. Therefore, it can be used to alleviate the condition of patients with severe COVID-19 in the short term (82). In conclusion, resveratrol, acetaminophen, estradiol, statins, dexamethasone, and quercetin may be potential drugs for the treatment of COVID-19.

In summary, we found that the biological processes in different organs after SARS-CoV-2 infection were heterogeneous. The invasion of SARS-CoV-2 causes multiple organ dysfunction through immune system disorders and cytokine storm syndrome. Moreover, the abnormal expression of hub genes (SRC, RHOA, CD40LG, CSF1, TNFRSF1A, FCER1G, ICAM1, LAT, LCN2, PLAU, CXCL10, CD40, IRF7, and B2M) was regarded as the main cause of cytokine storm syndrome in COVID-19 patients. Concurrently, we also found that resveratrol, acetaminophen, estradiol, statins, dexamethasone, and quercetin, etc. may be potential drugs for the treatment of COVID-19.

## Data Availability Statement

The original contributions presented in the study are included in the article/**Supplementary Material**. Further inquiries can be directed to the corresponding author.

## Author Contributions

MS, MZ, XCY, and XS conceived and designed the study. MZ, LYW, YQ, and XY contributed to data acquisition and data analysis. MZ wrote the original manuscript draft. XZ, XN, and LLW contributed to editing and revisions. MZ and XS contributed equally to this work. All authors contributed to the article and approved the submitted version.

## Funding

This work was supported by grants from Jilin Science and Technology Funds (20190201062JC) and the Education Department of Jilin Province (JJKH20190100KJ).

## Conflict of Interest

The authors declare that the research was conducted in the absence of any commercial or financial relationships that could be construed as a potential conflict of interest.

## Publisher’s Note

All claims expressed in this article are solely those of the authors and do not necessarily represent those of their affiliated organizations, or those of the publisher, the editors and the reviewers. Any product that may be evaluated in this article, or claim that may be made by its manufacturer, is not guaranteed or endorsed by the publisher.
