# Supplementary material for: The Molecular Mechanism of Multiple Organ Dysfunction and Targeted Intervention of COVID-19 Based on Time-Order Transcriptomic Analysis
Source: Front Immunol. 2021 Aug 24;12:729776. doi: 10.3389/fimmu.2021.729776 (PMC8421734; doi:10.3389/fimmu.2021.729776)
Supplement: Supplementary file 1 [file DataSheet_1.zip › Supplementary Material/Supplementary_Material.docx]

Supplementary Material

**Supplementary Figure 1.** DEG distribution results in the **(A)** brain, **(B)** lung, **(C)** trachea, **(D)** olf, and **(E)** smint at different time.

**Supplementary Table 1.** Data preprocessing.

**Supplementary Table 2.** DEG distribution results of different organs.

**Supplementary Table 3.** The biological significance of each organ in the time-order gene modules was explored by the Gene Ontology (GO) enrichment analysis.

**Supplementary Table 4.** Hub genes in the lung, trachea, and olf.

**Supplementary Table 5.** Potential drugs for COVID-19 treatment using the data from the comparative toxicogenomics database (CTD).
