# Supplementary figures and images for: The Molecular Mechanism of Multiple Organ Dysfunction and Targeted Intervention of COVID-19 Based on Time-Order Transcriptomic Analysis
Source: Front Immunol. 2021 Aug 24;12:729776. doi: 10.3389/fimmu.2021.729776 (PMC8421734; doi:10.3389/fimmu.2021.729776)

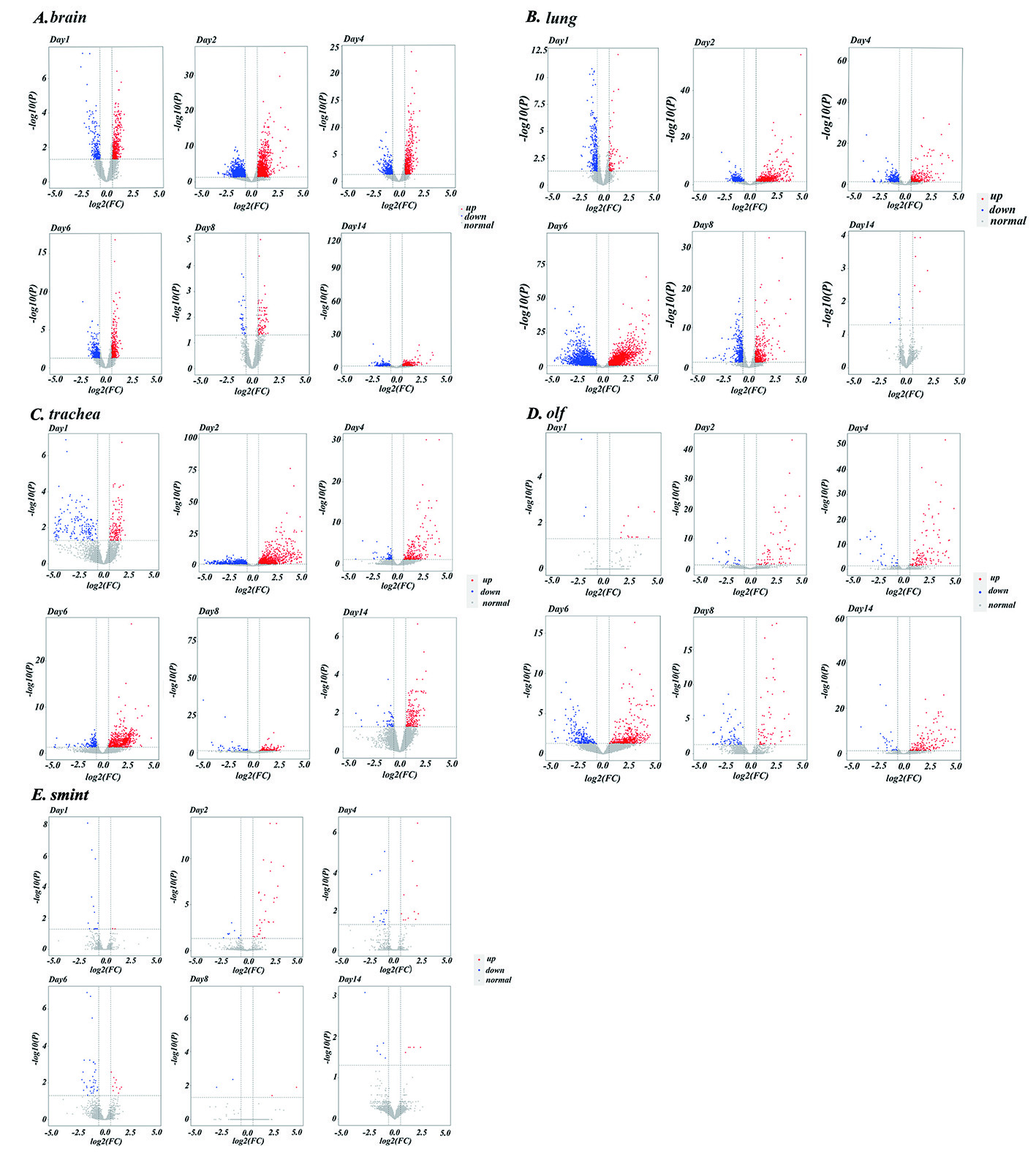

Supplement: Supplementary file 1 [file DataSheet_1.zip › Supplementary Material/Supplementary Fig 1.jpg]
